# Further development in the assessment of psychological flexibility: validation of the German committed action questionnaire

**DOI:** 10.1186/s12955-020-01497-8

**Published:** 2020-08-03

**Authors:** Yannik Terhorst, Harald Baumeister, Lance M. McCracken, Jiaxi Lin

**Affiliations:** 1 Department of Research Methods, Institute of Psychology and Education, Ulm University, Albert-Einstein-Allee 47, 89069 Ulm, Germany; 2 Department of Clinical Psychology and Psychotherapy, Institute of Psychology and Education, Ulm University, Lise-Meitner-Str. 16, 89081 Ulm, Germany; 3 Department of Psychology, Uppsala University, Box 1225 751 42, Uppsala, Sweden; 4 Institute of Sport and Sport Science, University Freiburg, Schwarzwaldstraße 175, 79117 Freiburg, Germany

**Keywords:** Committed action, Psychological flexibility, Chronic pain

## Abstract

**Background:**

Psychological flexibility is considered a fundamental aspect of health. It includes six interrelated facets: 1) cognitive defusion, 2) acceptance, 3) contact with the present moment, 4) self-as-context, 5) values, and 6) committed action. To gain further insight into psychological flexibility and its effects on health, reliable and valid instruments to assess all facets are needed. Committed action is one facet that is understudied. A long and short version of a validated measure (CAQ and CAQ-8) have been developed in English. Currently, there are no German versions of the CAQ. Aim of this study is to validate German-language versions of these in a chronic pain population.

**Methods:**

The CAQ instructions and items were translated and evaluated in a chronic pain population (*N* = 181). Confirmatory factor analysis and Mokken scale analysis were conducted to evaluate the German questionnaires. Correlations with health outcomes, including quality of life (SF-12), physical and emotional functioning (MPI, BPI, PHQ-9, GAD-7), pain intensity, and with other facets of psychological flexibility (CPAQ, FAH-II) were investigated for convergent validity purposes. Scale reliability was assessed by the alpha, MS, lambda-2, LCRC, and omega coefficient.

**Results:**

A bifactor model consisting of one general factor and two methodological factors emerged from the analysis. Criteria for reliability and validity were met. Medium to strong correlations to health outcomes and other facets of psychological flexibility were found. Results were similar to the original English version.

**Conclusions:**

The present study presents a valid and reliable instrument to investigate committed action in German populations. Future studies could expand the present findings by evaluating the German CAQ versions in non-pain populations. The role of committed action and the wider psychological flexibility model in pain and other conditions deserves further investigation.

## Background

Psychological flexibility is considered a fundamental aspect of health [1], and is defined as an ability to contact the present moment more fully as conscious human and to persist in or change behavior in accordance with one’s goals, values and what the situation’s affords [2]. In total, psychological flexibility consists of six interrelated facets: cognitive defusion, acceptance, contact with the present moment, self-as-context, values, and committed action [2]. The psychological flexibility model focuses on patterns of actions or behavior and has been identified as a key factor in many disorders and conditions (e.g. chronic pain, somatization, depression, anxiety, or psychological distress) [1–6].

Therapies such as Acceptance and Commitment Therapy (ACT) that focus on psychological flexibility have proven to be effective across several mental and somatic conditions (e.g. depression, anxiety and pain disorders) and settings (e.g. face-to-face, internet-based interventions); for reviews see [7–10]. However, in order to identify effective therapy components for specific patient populations as well as to better understand how therapy works [5, 11–14] it is important that key therapeutic processes of change can be measured reliably. Thus, measures have been developed or adopted to reflect psychological flexibility and its facets, including acceptance [15], present-focused awareness [16], cognitive defusion [17], self as context [18, 19] and values [20]. Several facets of psychological flexibility are well studied [5, 21–24]. However, the facet committed actions has not been investigated extensively [25].

Committed action is action guided by one’s goals and values, which is persistent in that it can incorporate discomfort and failure, and is flexible in that it can be stopped if it is unsuccessful [25, 26]. McCracken and colleagues developed a measure called Committed Actions Questionnaire (CAQ) [25] and a short version of this questionnaire CAQ-8 [26] in English to assess committed action. Both the long and the short version of the CAQ show satisfactory reliability and validity [25, 26]. To make the assessment of committed action widely accessible, translations and validations in other languages are needed. While the CAQ-8 provides a shorter and psychometrical sound assessment of committed action, a shorter item inventory is also accompanied by less content and potentially less information and details about the underlying behavior patterns [26]. Thus, validation of CAQ and CAQ-8 is needed. Currently, there are no German versions of the CAQ and CAQ-8.

The aim of this study is to validate German-language versions of the original version of the CAQ and the short version (CAQ-8) in a chronic pain population. The study examines the reliability and validity of both versions. In addition, based on item response theory (IRT), Mokken scaling analysis (MSA) will be applied to examine the dimensionality of the questionnaires and to determine whether the use of sum scores is adequate. Furthermore, associations between committed action and 1) the facet acceptance, 2) pain interference, 3) depression, 4) anxiety, 5) pain intensity, and 6) quality of life are investigated.

Construct validity of the original CAQ was supported by a latent two-factor and bifactor model, which fitted the data best [26]. We assume this to be equivalent for the CAQ-G. In terms of convergent and discriminate validity there is strong evidence for associations between committed action, mental health, pain-related symptoms, and other facets of psychological flexibility [25–32]: Based on these findings we expect medium positive correlations with health and medium to strong positive correlations to other facets of psychological flexibility.

## Methods

### Sample

The present sample consists of a subsample of individuals with chronic pain who participated in a three-armed randomized controlled trial (RCT) on the effectiveness of an online ACT based intervention called ACTonPain [33, 34]. Inclusion and exclusion criteria and the study procedure of ACTonPain are described in detail elsewhere [33, 34]. All participants gave informed consent for the use of their data for research purposes. The ACTonPain study was approved by the ethics committee of University Freiburg and registered at the German Clinical Trial Register (DRKS): DRKS00006183.

181 participants completed the CAQ-G at the 6 months follow-up of the ACTonPain study. Thus, *N* = 181 participants were included in the analyses. Participants were mainly women (86%). Average age was 51.97 years (SD = 13.12). The majority was employed (58%) and married (55%). Further descriptive characteristics of this subsample are summarized in Table 1.

**Table 1:** Descriptive characteristics (*N* = 181)

Variable	
Gender	86% female
Age	Mean (M) = 51.97 (sd = 13.12) years
Family status	Single 17%
	In relationship 15%
	Married 55%
	Divorced 7%
	Living separated 2%
	Widowed 4%
Education	Basic school qualification 14%
	Secondary school qualification 36%
	Technical college qualification 18%
	College qualification and higher 31%
Employment	Unemployed 4%
	In education or study 3%
	Employed 58%
	Retired 35%
Pain duration in weeks	Median (MD) = 60 (95% CI: 9–463)
	M = 110
Pain location	Head 14%
	Neck 11%
	Shoulder 6%
	Back 62%
	Other 35%

### Translation of the CAQ

We followed international recommendations for translating and back-translating the CAQ [35]. First, the CAQ was translated from the original to German by a German clinical psychologist (JL) fluent in English and with specific knowledge of the research area. The questionnaire was then back-translated by two Germans, one psychologist fluent in English and with experience in working with instrument validation (HB) and another who worked as a translator for both directions (PS). Thereafter, the translated and back-translated versions were evaluated by the author of the English language original (LM). Discrepancies were discussed and resolved by consensus.

The translated versions of the CAQ-G and CAQ-8-G are presented in the additional file 1.

### Measures

#### German committed action questionnaire

The German version of the committed action questionnaire (CAQ-G) is a translation of the English CAQ [25]. The 18-item version contains nine positive keyed items and nine negative keyed items. Responders have to indicate to which extent an item applies to them on a scale from 0 (never true) to 6 (always true). The reliability of the 18 item version was high (α = .91) [25]. Further correlation analyses in previous studies showed good convergent and divergent validity with other constructs (e.g. pain acceptance or emotional functionality) [25]. Items of the CAQ were reduced to eight for a short version [26]. Reliability of the CAQ-8 is good (α = .87) [26].

#### Physical and emotional functioning

In accordance with the Initiative on Methods, Measurement, and Pain Assessment in Clinical Trials (IMMPACT) recommendations [36, 37] the German Multidimensional Pain Inventory (MPI) and the German Brief Pain Inventory (BPI) were used to assess pain interference. The German MPI consists of 10 questions assessing interference on a behavioral (e.g. daily activities) and emotional level (e.g. ability to experience joy) with an excellent reliability (α = .94) [38]. Interference in physical functioning (e.g. walking ability), emotional functioning (e.g. mood) and sleep is assessed with seven items in the German BPI. Reliability of the German BPI is excellent with a reliability of α = .88 [39].

Depression was assessed with the German Patient Health Questionnaire (PHQ-9) [40]. The German Generalized Anxiety Disorder questionnaire (GAD-7) was used to assess anxiety [41, 42]. Both, the German PHQ-9 and the German GAD-7 show an excellent reliability of α = .89 [41, 43].

#### Health related quality of life

To assess health-related quality of life the Short Form 12 (SF12) was used [44]. The SF-12 is divided into physical (SF12 PCS) and mental health (SF12 MCS) and covers eight health domains: physical functioning, role limitations, pain, general health perception, vitality, mental health, emotional role and social functioning [44]. The physical subscale (α = .77) and mental health subscale (α = .80) have good reliability [44].

#### Pain intensity

As recommended by the IMMPACT pain intensity was assessed with a 11 point numerical rating scale (NRS), on which responders indicated average pain during the last week from 0 to 10, with 0 = “no pain” and 10=“pain as bad as you can imagine” [36, 37].

#### Psychological flexibility

To assess psychological flexibility, the German version of the Acceptance and Action Questionnaire — II (AAQ-II [45] German: Fragebogen zu Akzeptanz und Handeln II (FAH-II [46]) was used. This questionnaire consists of 7 items and shows good to excellent psychometric properties in a German sample [46]. On a 7-point scale that ranges from 0 = “never true” to 6 = “always true”, participants rate processes of experiential avoidance and psychological inflexibility. Reliability of the FAH-II is good (α = .84) [46].

In addition to the FAH-II, the German version of Chronic Pain Acceptance Questionnaire was used (CPAQ-D) [47]. On a 7-point scale that ranges from 0 = “never true” to 6 “always true”, participants rate their activity engagement (CPAQ-AE) and pain willingness (CPAQ-PW) on 20 items. Reliability of the German CPAQ and its subscales is good (α = .84 to .87) [47].

### Analyses

Analyses were divided in three parts: First, confirmatory factor analysis was used to evaluate the latent structure of the CAQ-G. Second, Mokken Scale Analysis was conducted to further evaluate the scalability and the adequateness of sum scores of the CAQ-G and CAQ-8-G. In a third step correlations between the short version, the long version, acceptance (FAH-II, CPAQ), depression (PHQ-9), anxiety (GAD-7), pain interference (MPI, BPI), pain intensity (NRS), and quality of life (SF12) were calculated.

#### Confirmatory factor analysis: construct validity

To examine the latent structure of the 18 items of the CAQ, a confirmatory factor analysis (CFA) was used. Based on the theoretical background and the results of the initial CAQ and the 8 item CAQ [25, 26, 48, 49] three competing models were derived. The first is a single factor model, representing a general committed action (CA) construct. The second is a two factor model representing (co)variances of positive or negative keyed items. Correlation between the two factors was allowed. The third is a bifactor model including three latent factors, a general factor representing the CA construct as in model 1 and two item class specific factors accounting for (co)variance of positive and negative keyed items, respectively. Correlations between factors were set to zero in the bifactor model. Values of negative keyed items were reversed for analyses.

Model fit of the three models were determined by Chi-square statistic. Due to the high sensitivity of chi-square, additional fit indices (RMSEA as a non-centrality parameter, SRMR as a residual fit index and CFI and TLI as incremental indices) were used if significance occurs. Based on standard criteria for model fit a RMSEA ≤ .05, a CFI > .95, a TLI > .95 and SRMR ≤ .06 indicates a good model fit [50].

A check for multivariate normality revealed that neither multivariate normality nor normality for single item was given. Thus, and to be consistent with later Mokken Scale Analysis (MSA) multivariate normality assumption and assumption of interval scale level of items were rejected. Instead of interval level, ordinal scale level was assumed. In case of non-normal ordered data diagonal weighted least square (DWLS) estimator is the most accurate estimator [51, 52] and therefore was used in CFA.

##### Convergent validity

Convergent validity will be assessed via correlations to other constructs/ questionnaires. Medium absolute correlations (> I.5I) to psychological flexibility, acceptance and small to medium absolute correlation (> I.2I) with emotional and physical functioning would indicate good validity.

#### Mokken scale analysis

As done in the development of the original CAQ-8 [26] Mokken Scale Analysis (MSA) was conducted to evaluate the CAQ-8-G. In short, MSA is a scaling technique for ordinal data. Frequently, MSA is used for scaling test and it is related to nonparametric item response theory (IRT) [53]. MSA allows both, selecting items for scales and testing of assumptions of nonparametric IRT [53].

The key parameter in MSA is Loevinger’s H – where the scaling parameter for item i is H_i_ and the overall scalability of all items clustering onto scale k is H_k_. H_i_ indicates the strength of the relationship between a latent variable (committed action in this case) and item i. High scalability indicates whether the probability to score higher on item i increases with an increase on the latent variable. As a rule of thumb, a scale is considered weak if H < .4, moderate if .4 ≤ H ≤ .5 and strong if H > .5 [54]. For detailed introduction see [53, 55, 56].

MSA assumes item monotonicity. Monotonicity was evaluated using item-rest regression [54, 57]. *crit* statistic was used to check for meaningful violations (*crit* > 40) [57, 58]. Further non-intersection is assumed. Non-intersection was evaluated by the *restscore* method (no violation: *crit* < 40, minor violations: *crit* 40–80, serious violations: *crit* > 80) [53, 55, 57, 58].

#### Reliability

As recommended by van der Ark [55] reliability of scales will be assessed by Cronbach’s alpha [59], Molenaar-Sijtsma method (MS) [60, 61], lambda-2 [62] and latent class reliability coefficient (LCRC) [63]. In addition, we calculated omega (ω), since it provides a more unbiased estimation of reliability than the widely used Cronbach’s alpha [64–66]. Bootstrapped and bias corrected confidence intervals was obtained for ω using the procedure introduced by Zhang and Yuan [67].

#### Analysis software

For all analyses, we used the software R [68]. For CFA the R package “lavaan” (version: 0.5–23.1097) was used [69]. MSA was conducted with the R package “mokken” [53, 55]. Correlations were calculated using the “psych” package (version: 1.7.8.) [70]. For omega the “coefficientalpha” package was used [67]. The R script used for analysis and the anonymized data set can be requested from corresponding author (see availability of data and materials for further details).

## Results

### Missingness and missing data handling

Missingness only occurred in the descriptive variable pain duration. A total of 6 responses (3.3%) were missing. For the calculation of pain duration missing values were excluded.

### CFA: construct validity

Three competing models were tested. First, a one factor model (all items only measure the construct Committed Action [CA]), second a two factor model (items measure one of two factors depending on how items are keyed) and third a bifactor model (CA factor plus two item class specific factors). Only the bifactor model resulted in an acceptable fit based on RMSEA, SRMR, CFI and TLI (see Table 2).

**Table 2:** Confirmatory factor analysis – comparison of models

	Noncentrality parameters					Residual fit indices	Incremental indices	
Modell	Chi-square	df	*p* value	RMSEA	90% CI	SRMR	CFI	TLI
One factor	791.37	135	<.001	.16	.15–.18	.10	.98	.98
Two factor	332.78	134	<.001	.09	.08–.10	.06	.99	.99
Bifactor	173.22	117	<.001	.05	.04–.07	.05	.99	.99

The construct validity is supported by the bifactor model. The fit of the bifactor model indicates that items measure a common latent construct (CA) as well as item class specific factors (=methodological factors of positive and negative keyed items). Loadings on the CA factor in the bifactor are presented in Table 3.

**Table 3:** Factor loadings on latent committed action variable based on bifactor model

Item	Standardized loading on CA	*P* value^1^	Included in short scale
01	0.711	<.001	
02	0.941	<.001	✓
03	0.950	<.001	✓
04	0.897	<.001	✓
05^n^	0.126	.472	
06	0.811	<.001	
07	0.876	<.001	✓
08^n^	0.612	<.001	✓
09^n^	0.436	<.001	✓
10^n^	0.447	<.001	
11^n^	0.349	.023	
12	0.670	.014	
13	0.693	.004	
14^n^	0.453	<.001	
15	0.656	<.001	
16^n^	0.412	<.001	
17^n^	0.504	<.001	✓
18^n^	0.484	<.001	✓

Note: ^1^*p*-values were obtained using bootstrap; ^n^ indicates negative keyed items;

### Mokken scale analysis

MSA for the CAQ-G resulted in a poor scalability (H = .391 [se = .031]). Assumption of monotonicity was met, while non-intersection was highly violated (12 minor and 6 critical violations). Reliability was high (α = .91 [95% CI: .89 to .93], MS = .91, lambda.2 = .92, LCRC = .91, ω = .91 [95% CI: .88 to .93]). Scalability for all positive items was strong (H = .643 [se = .039]). Monotonicity was met and evaluation of non-intersection yielded only two minor violation (crit_item4_ = .40, *crit*_*item15*_ = 43). Reliability of the positive subscale was excellent (α = .93 [95% CI: .92 to .95), MS = .94, lambda.2 = .93, LCRC = .92, ω = .93 [95% CI: .91 to .95]). Negative subscale showed poor scalability (H = .365 [se = .033]), no violations of monotonicity and four minor and one serious violation of non-intersection. Reliability was good (α = .82 [95% CI: .79 to .86], MS = .83, lambda.2 = .83, LCRC = .83, ω = .83 [95% CI: .78 to .86]).

The CAQ-8-G, a scale with the corresponding items of the English CAQ-8 was tested with MSA in addition to the CAQ-G. This scale was medium to strong (H = .453 [se = .038]) with high reliability (α = .85 [95% CI: .82 to .89), MS = .86, lambda.2 = .86, LCRC = .86, ω = .85 [95% CI: .79 to .88]). Assumption of monotonicity was met. Seven items showed minor violations of non-intersection.

### Convergent validity

To assess convergent validity of the scale, correlations with other measures were calculated. As assumed the CAQ-G has good convergent validity: The CAQ-G scales demonstrated high correlations with other measures of psychological flexibility or facets of psychological flexibility (CPAQ and FAH). Further, medium correlations to indicators for physical and emotional functioning (MPI-interference, BPI-interference, PHQ, GAD) as well as to quality of life (SF12 MCS) were found. No correlation with pain intensity (NRS) and SF12 PCS was found. See Table 4 for correlations.

**Table 4:** Correlation analysis: convergent validity

	1	2	3	4	5	6	7	8	9	10	11	12	13	14	15
**1 CPAQ**_**AE**_	1^*^														
**2 CPAQ**_**PW**_	.59^*^	1^*^													
**3 CPAQ**_**total**_	.93^*^	.85^*^	1^*^												
**4 FAH II**	−.59^*^	−.65^*^	−.69^*^	1^*^											
**5 MPI - interference**	−.63^*^	−.46^*^	−.63^*^	.45^*^	1^*^										
**6 BPI - interference**	−.51^*^	−.4^*^	−.52^*^	.43^*^	.76^*^	1^*^									
**7 PHQ**	−.5^*^	−.49^*^	−.55^*^	.64^*^	.58^*^	.61^*^	1^*^								
**8 GAD**	−.39^*^	−.48^*^	−.48^*^	.68^*^	.39^*^	.42^*^	.78^*^	1^*^							
**9 NRS**	−.38^*^	−.23^*^	−.35^*^	.22^*^	.73^*^	.77^*^	.43^*^	.22^*^	1^*^						
**10 SF12**_**PCS**_	.34^*^	.19	.31^*^	−.05	−.54^*^	−.48^*^	−.2	.01	−.55^*^	1^*^					
**11 SF12**_**MCS**_	.53^*^	.5^*^	.58^*^	−.76^*^	−.47^*^	−.45^*^	−.77^*^	−.74^*^	−.22^*^	−.12	1^*^				
**12 CAQ-8-G**	.54^*^	.48^*^	.58^*^	−.67^*^	−.31^*^	−.28^*^	−.48^*^	−.49^*^	−.12	.02	.57^*^	1^*^			
**13 CAQ-G**_**Neg**_	.39^*^	.43^*^	.45^*^	−.63^*^	−.22^*^	−.2	−.38^*^	−.48^*^	−.04	.01	.48^*^	.84^*^	1^*^		
**14 CAQ-G**_**Pos**_	.54^*^	.46^*^	.56^*^	−.56^*^	−.30^*^	−.28^*^	−.43^*^	−.40^*^	−.11	.02	.53^*^	.86^*^	.55^*^	1^*^	
**15 CAQ-G**	.53^*^	.51^*^	.58^*^	−.67^*^	−.30^*^	−.28^*^	−.46^*^	−.50^*^	−.09	.02	.57^*^	.96^*^	.87^*^	.89^*^	1^*^

Note: ^*^ indicates significance below *p* ≤ .05 (adjusted for multiple testing); *MPI* Multidimensional Pain Inventory, *BPI* Brief Pain Inventory, *PHQ 9* Patient Health Questionnaire (9 items version), *GAD 7* Generalized Anxiety Disorder questionnaire (7 item version), *NRS* Numeric Rating Scale of Pain Intensity, *SF12_PCS* Health related quality of life the Short Form 12 (physical), *SF12_MCS* Health related quality of life the Short Form 12 (mental health), *FHA II* German version of the Acceptance and Action Questionnaire – II (AAQ-II), low values indicate high flexibility, *CPAQ AE* Chronic Pain Acceptance Questionnaire activity engagement scale, *CPAQ PW* Chronic Pain Acceptance Questionnaire pain willingness scale, *CPAQ Total* Chronic Pain Acceptance Questionnaire, *CAQ-G* German version of the committed action questionnaire, CAQ-G_neg_ includes only negative keyed items, CAQ-G_pos_ includes only positive keyed items, *CAQ-8-G* German version of the English short version of the CAQ

## Discussion

Aim of this study was to validate the CAQ-G and CAQ-8-G to provide a comprehensive assessment of committed action (CA) in German. Overall, the findings within this study are similar to the results of the English CAQ and CAQ-8 [25, 26]. Construct validity of the CAQ-G was demonstrated by the bifactor model. Expectations regarding convergent validity were met. Reliability was high. While MSA for the positive subscale of the CAQ-G and the CAQ-8-G indicate that the use of sum scores is adequate, sum score of the negative subscale should be used with caution due to major violations.

As shown in the validation of the original CAQ and assumed for the CAQ-G, a bifactor model explains the latent structure of the CAQ best [25, 26]. Given the theoretical background of the CAQ it can be assumed that the general factor represents the latent construct committed action, while the other factors explain covariance introduced by methodology (e.g. positive and negative wording of items). While the construct validity is demonstrated by the present results, it also has to be highlighted that also two-factor model was fitting the original CAQ [26]. This is not true for the CAQ-G, since the RMSEA exceeded the pre-defined cut-off. Future studies using the CAQ-G should investigate whether this is an artifact of the present study or a robust finding. However, based on the present findings only a the bifactor model confirms the construct validity of the CAQ-G.

While CFA confirms the assumed structure, it also highlights that some items are poor or no meaningful indicators for the general latent factor CA (e.g. item 05: loading = .13, *p* = .472) and only good indicators for the methodological factors. Unsurprisingly MSA shows that the scalability of the total scale including the poor indicators is weak. Thus, a short version only including strong indicators would offer a more reliable assessment of CA. MSA demonstrates that the CAQ-8-G is such a scale. Similar to the original CAQ-8 [26], the CAQ-8-G showed medium to strong scalability and excellent reliability. This is also true for the positive subscale of the CAQ, but not for the negative subscale. Hence, only the CAQ-8-G or the positive subscale of the CAQ should be used in practice.

In terms of convergent validity, the present results almost perfectly mirror the results from the original CAQ validation and the hypothesis regarding the correlations were met. Especially, The correlations of the CAQ-G and CAQ-8-G with variables of psychological flexibility, emotional and physical functioning are almost identical to the correlations of the English versions (r_CPAQ_ = .49, r_PHQ-9_ = −.57, r_SF-36-Mental_ = .58) [25]. Overall, the medium correlations between psychological flexibility, facets of psychological flexibility, emotional and physical functioning are in line with many prior studies [25, 27–32]. Correlations with variables of emotional and physical functioning as well as with quality of life indicate that psychological flexibility as wider process, or committed action specifically, might act as mechanisms of change in treatments of chronic pain. As such measures of these processes may help in the further development of psychological treatments for chronic pain.

Although the present results are promising, a few limitations have to be taken into account for interpretation. All analyses are based on a single sample of 181 German individuals with chronic pain from an RCT evaluating the effectiveness of an internet-based intervention [33, 34]. Although individuals show a variety in socio-demographic backgrounds and in pain related variables such as pain duration of pain location, the present sample is not a normative sample representative for the general population. Hence, it is unclear whether similar results would emerge in other pain populations, population with different somatic or mental health conditions, or healthy populations. Thus, the CAQ-G and CAQ-8-G should be validated in additional populations to examine generalizability.

Moreover, the reduced items in the CAQ-8-G might lead to loss of information and the construct of CA is less comprehensively covered. However, the high correlation between the CAQ-G and the CAQ-8-G (r = .96) indicate a rather low and maybe insignificant loss of information. Thus, the gains of time efficiency and loss of information should be weighed for each individual study.

Lastly a limitation emerges of the applied definition of committed action itself. As highlighted by McCracken and colleagues [26] the definition of committed action applied in the English CAQ and CAQ-8 and the respective German versions may seem somewhat narrow: For instance, behavior change with no difficulties or integration of behavior change into generalized patterns are not covered by the CAQ. Future studies might target this limitation and expand the current definition by developing new items. However, based on the high and medium correlations to health and psychological flexibility constructs it can be assumed that the current CAQ already covers relevant aspects of committed action.

## Conclusion

With the development and examination of the CAQ-G and CAQ-8-G the present study provides a new way to assess patterns of actions and behavior in German populations. Construct validity and convergent validity of the CAQ-G and the CAQ-8-G was good. However, taking the scalability into account, only the CAQ-8-G and the positive subscale of the CAQ-G should be used for a valid and reliable assessment of CA in German populations. Furthermore, the present results highlight the close relationship between psychological flexibility and health. To develop a deeper understanding of the mechanisms behind effective therapy and the causal relationships between CA and health, further studies are needed.

## Supplementary information

**Additional file 1.** Committed Action Questionnaire.

## Data Availability

The dataset analyzed during the current study is not publicly available due to the sensitive nature of patients’ data. Participants agreed on the use of their anonymized data for research purposes only. An anonymized version of the dataset with no direct or indirect identifiers can be provided by HB on reasonable request, in which clear research and no other purposes are stated. The R script used for all analyses can be provided on request by YT. Data requestors will need to sign a data access agreement. Provision of data is subject to data security regulations. Investigator support depends on available resources.

## Abbreviations

ACT: Acceptance and Commitment Therapy
CAQ: Committed Actions Questionnaire
CAQ-8: Short version of Committed Actions Questionnaire
IRT: Item Response Theory
MSA: Mokken Scaling Analysis
RCT: Randomized Controlled Trial
DRKS: German Clinical Trial Register
CAQ-G: German Committed Action Questionnaire
CAQ-8-G: Short version of German Committed Actions Questionnaire
IMMPACT: Initiative on Methods, Measurement, and Pain Assessment in Clinical Trials
MPI: Multidimensional Pain Inventory
BPI: Brief Pain Inventory
PHQ-9: Patient Health Questionnaire
GAD-7: Generalized Anxiety Disorder questionnaire
SF12: Health-related quality of life Short Form 12
SF12 PCS: Divided into physical
SF12 MCS: Mental health
AAQ-II: Acceptance and Action Questionnaire — II
FAH-II: Fragebogen zu Akzeptanz und Handeln II
CPAQ-D: Chronic Pain Acceptance Questionnaire
CPAQ-AE: Activity Engagement
CPAQ-PW: Pain Willingness
NRS: Numerical Rating Scale
CFA: Confirmatory Factor Analysis
CA): Committed Action
DWLS: Diagonal Weighted Least Square
MS: Molenaar-Sijtsma method
LCRC: Latent Class Reliability Coefficient
