# Supplementary material for: Further development in the assessment of psychological flexibility: validation of the German committed action questionnaire
Source: Health Qual Life Outcomes. 2020 Aug 3;18:260. doi: 10.1186/s12955-020-01497-8 (PMC7397648; doi:10.1186/s12955-020-01497-8)
Supplement: Supplementary file 1 — Additional file 1. Committed Action Questionnaire. [file 12955_2020_1497_MOESM1_ESM.docx]

Committed Action Questionnaire

|  | Below you will find a list of statements. Please rate the truth of each statement as it applies to you by circling a number. Use the following rating scale to make your choices. For instance, if you believe a statement is “Always True”, you would circle the 6 next to that statement. | Nachfolgend finden Sie eine Liste von Aussagen. Bitte schätzen Sie ein, wie sehr jede dieser Aussagen auf Sie zutrifft, indem Sie eine der nebenstehenden Nummern markieren. Verwenden Sie die folgende Bewertungsskala, um Ihre Wahl zu treffen. Wenn zum Beispiel eine Aussage "immer" zutrifft, würden Sie die 6 auswählen. |
| --- | --- | --- |
|  | \| **0** \|  \| **1** \|  \| **2** \|  \| **3** \|  \| **4** \|  \| **5** \|  \| **6** \| \| --- \| --- \| --- \| --- \| --- \| --- \| --- \| --- \| --- \| --- \| --- \| --- \| --- \| \| **Never** **True** \|  \| **Very Rarely** **True** \|  \| **Seldom** **True** \|  \| **Sometimes True** \|  \| **Often True** \|  \| **Almost Always True** \|  \| **Always True** \| \| **trifft nie zu** \|  \| **trifft fast nie zu** \|  \| **trifft selten zu** \|  \| **trifft manchmal zu** \|  \| **trifft häufig zu** \|  \| **trifft fast immerzu** \|  \| **trifft immer zu** \| | |
| **#** | **CAQ** | **CAQ-G** |
| 1 | I am able to persist with a course of action after experiencing difficulties. | Ich kann eine Handlung fortsetzen, auch wenn ich Schwierigkeiten erfahre. |
| 2 | When I fail in reaching a goal, I can change how I approach it. | Wenn ich es nicht schaffe, ein Ziel zu erreichen, kann ich meine Herangehensweise zur Zielerreichung ändern. |
| 3 | I can remain committed to my goals even when there are times that I fail to reach them. | Ich kann meine Ziele weiter verfolgen, auch wenn es Zeiten gibt, in denen ich sie nicht erreiche. |
| 4 | When a goal is difficult to reach, I am able to take small steps to reach it. | Wenn ein Ziel schwer zu erreichen ist, kann ich kleine Schritte gehen, um es zu erreichen. |
| 5 | I act impulsively when I feel under pressure. | Ich handle impulsiv, wenn ich mich unter Druck fühle. |
| 6 | I prefer to change how I approach a goal rather than quit. | Ich ändere lieber meine Herangehensweise, ein Ziel zu erreichen als aufzugeben. |
| 7 | I am able to follow my long term plans including times when progress is slow. | Ich kann meine langfristigen Pläne auch in Zeiten langsamer Fortschritte verfolgen. |
| 8 | When I fail to achieve what I want to do, I make a point to never do that again. | Wenn ich nicht das erreiche, was ich tun möchte, beschließe ich, es nie mehr wieder zu tun. |
| 9 | I get stuck doing the same thing over and over even if I am not successful. | Ich verrenne mich darin, dieselben Dinge immer wieder zu machen, auch wenn ich damit erfolglos bin. |
| 10 | I find it difficult to carry on with an activity unless I experience that it is successful. | Ich finde es schwierig, an einer Aktivität dranzubleiben, es sei denn ich merke, dass es erfolgreich ist. |
| 11 | I am more likely to be guided by what I feel than by my goals. | Ich werde eher durch meine Gefühle als durch meine Ziele geleitet. |
| 12 | I am able to pursue my goals both when this feels easy and when it feels difficult. | Ich kann meine Ziele weiterverfolgen, egal ob es sich einfach oder schwierig anfühlt. |
| 13 | I am able to persist in what I am doing or to change what I am doing depending on what helps me reach my goals. | Ich kann das, was ich tue, fortführen oder verändern, je nachdem, was mir dabei hilft, meine Ziele zu erreichen |
| 14 | If I make a commitment and later fail to reach it, I then drop the commitment. | Wenn ich mich für eine Sache verpflichte, die ich im weiteren Verlauf nicht erreiche, lasse ich die Verpflichtung fallen. |
| 15 | I am able to incorporate discouraging experiences into the process of pursuing my long term plans. | Ich kann entmutigende Erfahrungen als Teil des Prozesses sehen, meine langfristigen Pläne zu verfolgen. |
| 16 | If I feel distressed or discouraged, I let my commitments slide | Wenn ich mich gestresst oder entmutigt fühle, lasse ich meine Verpflichtungen schleifen. |
| 17 | I get so wrapped up in what I am thinking or feeling that I cannot do the things that matter to me. | Ich werde von meinen Gedanken und Gefühlen so sehr eingenommen, dass ich die Dinge nicht tun kann, die mir wichtig sind. |
| 18 | If I cannot do something my way, I will not do it at all. | Wenn ich etwas nicht auf meine Art und Weise tun kann, werde ich es überhaupt nicht tun. |
